# Verticillium Wilt of Mint in the United States of America

**DOI:** 10.3390/plants9111602

**Published:** 2020-11-18

**Authors:** Jeremiah K. S. Dung

**Affiliations:** Central Oregon Agricultural Research and Extension Center, Department of Botany and Plant Pathology, Oregon State University, Madras, OR 97741, USA; jeremiah.dung@oregonstate.edu

**Keywords:** *Verticillium dahliae*, *Mentha*, soilborne plant pathogens, disease management

## Abstract

Verticillium wilt, caused by the fungus *Verticillium dahliae,* is the most important and destructive disease of mint (*Mentha* spp.) in the United States (U.S.). The disease was first observed in commercial mint fields in the Midwestern U.S. in the 1920s and, by the 1950s, was present in mint producing regions of the U.S. Pacific Northwest. Verticillium wilt continues to be a major limiting factor in commercial peppermint (*Mentha* x *piperita*) and Scotch spearmint (*Mentha* x *gracilis*) production, two of the most important sources of mint oil in the U.S. The perennial aspect of U.S. mint production, coupled with the soilborne, polyetic nature of *V. dahliae*, makes controlling Verticillium wilt in mint a challenge. Studies investigating the biology and genetics of the fungus, the molecular mechanisms of virulence and resistance, and the role of soil microbiota in modulating host-pathogen interactions are needed to improve our understanding of Verticillium wilt epidemiology and inform novel disease management strategies. This review will discuss the history and importance of Verticillium wilt in commercial U.S. mint production, as well as provide a format to highlight past and recent research advances in an effort to better understand and manage the disease.

## 1. Mint Production in the United States

The genus *Mentha* L. (family Lamiaceae) encompasses a group of aromatic, mostly perennial herbs that are used in medicines, flavors, and fragrances [1,2]. The aromatic properties of mints are derived from their essential oils, which are produced on leaves in glandular trichomes and extracted using steam distillation [1,3]. *Mentha* oils are commonly used to flavor foods, gum, confectionaries, dentifrices, and liqueurs, and are also used in pharmaceuticals, cosmetics and aromatherapy products. There are several economically important species and hybrids that are grown commercially for their essential oils, and many more that are grown for local consumption [1].

The cultivation of mint for oil has a long history in the United States, where commercial peppermint (*Mentha* x *piperita* L.) production and distillation was first documented in western Massachusetts in the 1790s [1,4]. By the 1830s, production moved into New York and Michigan, where most of the production occurred until the end of the nineteenth century [4]. By the late 1800s, growers were cultivating and distilling oil from native spearmint (*Mentha spicata* L.), Scotch spearmint (*Mentha* x *gracilis* Sole), and ‘Black Mitcham’ peppermint, the latter being a very productive variety of peppermint from England with highly desirable oil qualities [1,4]. The early 20th century saw production shift into the muck soils of Michigan and Indiana, and further west into the Pacific Northwest states of Oregon, Washington, and Idaho, where the majority of U.S. mint oil is currently produced [1,4]. A smaller acreage of mint is also cultivated in the United States to provide dried leaf for teas. Since 2000, the average annual production of peppermint and spearmint (both *M*. x *gracilis* and *M*. *spicata*) oil in the United States was 2,866,567 kg (on 28,566 ha) and 1,086,852 kg (on 8170 ha), respectively, with a combined average annual farmgate of USD 144,120,150 [5]. India and China are also major global producers of mint oil, but mint production in these countries is focused on *M. arvensis* oil [1].

In 1924, a disease of unknown etiology was observed on a peppermint farm in Mentha, Michigan [4,6]. Later identified as Verticillium wilt, the disease soon spread into other production fields in the southwest area of the state. In response, infested fields in the area were abandoned from mint cultivation and production shifted to central Michigan. Consequently, Verticillium wilt was subsequently reported in Indiana [7], Oregon [8], and Washington [9]. By the mid-twentieth century, Verticillium wilt was the major production concern facing the U.S. mint industry [4].

## 2. Verticillium Wilt of Mint

### 2.1. Symptoms in Mint

Nearly 100 years after it was first identified in peppermint, Verticillium wilt continues to be the most important and destructive disease affecting commercial mint oil production in the United States [2,10,11,12,13]. *Verticillium* spp. have also been reported on mint in Brazil, Bulgaria, Canada, Egypt, Venezuela, and Zimbabwe [14,15], but the disease is of primary importance in U.S. mint production. Two of the three major cultivars of mint grown in the U.S., ‘Black Mitcham’ peppermint and Scotch spearmint, are highly susceptible to Verticillium wilt, while native spearmint is moderately to highly resistant [16,17]. Cornmint, which is the major *Mentha* oil crop in India and China, exhibits moderate resistance to Verticillium wilt [17].

**Figure 1:**
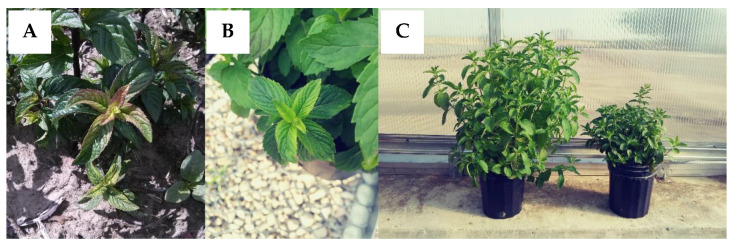
(**A**) Twisted, curled, and crescent-shaped leaves of peppermint and (**B**) Scotch spearmint. (**C**) Verticillium wilt can cause severe stunting in susceptible cultivars.

**Figure 2:**
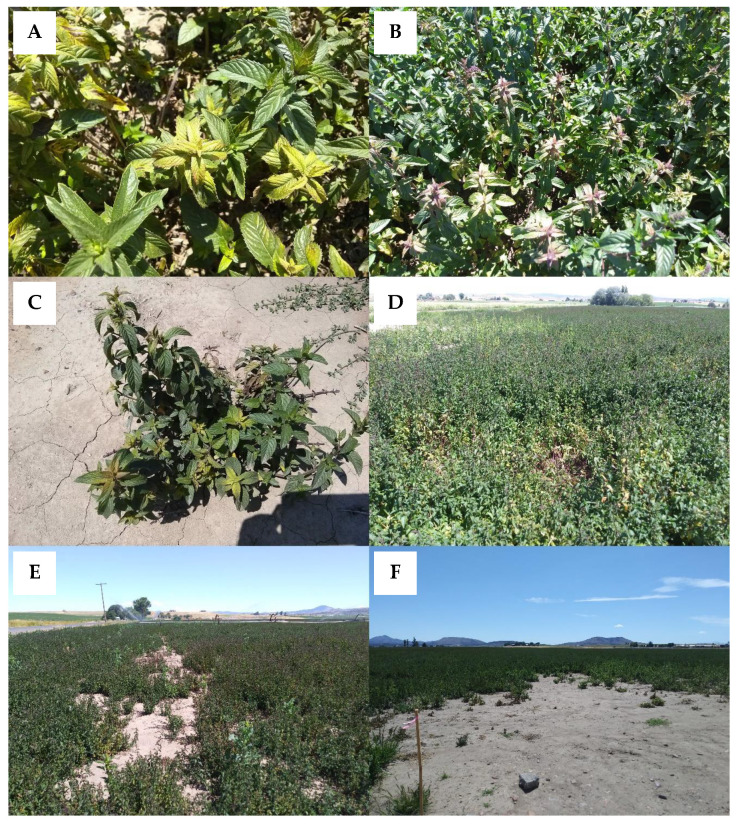
(**A**) Chlorosis and (**B**) anthocyanescence in peppermint caused by Verticillium wilt. (**C**) Verticillium wilt symptoms on an individual peppermint plant and (**D**) in the field. (**E**) The disease can result in bare patches in fields and (**F**) are often more pronounced at field entry points.

Symptoms of Verticillium wilt in mint can vary depending on the Mentha species that is infected, the stage of the crop, and environmental conditions. While symptoms do not often follow a regular progression, in most cases symptoms begin in the apical meristem, namely the asymmetric development of leaves that appear to twist and curl into a crescent shape (Figure 1A,B). Internodes can be shorter, resulting in the bunching of upper leaves and/or stunted plants (Figure 1C). New growth can be chlorotic or exhibit reddening or bronzing (Figure 2A,B). As the disease progresses in peppermint, chlorosis develops at the base of the plant and progresses upwards, resulting in defoliation of lower leaves; in spearmint, chlorosis may be more pronounced and affect the whole plant. Symptoms can occur on one or a few stems or the entire plant depending on the extent and duration of infection (Figure 2C). Infected stems may exhibit xylem discoloration. Susceptible mints that succumb to the disease eventually exhibit wilt and senesce prematurely [18]. Flowering or other stresses can expedite wilt and plant senescence. Over time, foci of dead plants develop (Figure 2D) and coalesce, leaving large areas of mint fields bare (Figure 2E).

### 2.2. Economic Impacts

Economic losses due to Verticillium wilt of mint are both primary and secondary [6]. Primary losses are realized as reduced oil yields caused by stunting, defoliation, or early senescence. The cumulative effects of disease symptoms and reduced photosynthetic potential can weaken stands going into winter, resulting in increased winter kill and poor regrowth the following spring [19].

Secondary losses are manifested in several ways. Disease foci often lead to bare patches that are prone to weed infestation which, if distilled along with the mint, can adulterate the oil profile and leave it less desirable [1]. Infested fields can be fumigated to return them to profitable mint production, but this practice is expensive and not always possible to perform due to increasing health, environmental, economic, or regulatory concerns. Growers may choose to produce a more resistant cultivar of peppermint or spearmint; however, these varieties often yield less than the susceptible ‘Black Mitcham’ cultivar, and there is typically less demand for their oils due to their limited end-use or competition from international markets.

Mint is a perennial crop and, in the absence of Verticillium wilt, many areas could keep stands of mint in production for up to 5 years, and in some cases even up to 20 years [6]. In the presence of Verticillium wilt, the stand life of peppermint is typically three years or less, though this can depend on the level of soil infestation, the level of cultivar resistance, environmental conditions, and economic considerations. Heavily infested fields are often abandoned from mint production, which represents a loss of productive cropland available for future mint cultivation.

## 3. The Pathogen: *Verticillium dahliae*

The causal agent of Verticillium wilt, *Verticillium dahliae* (Kleb.), is a soilborne Ascomycete with a worldwide distribution in temperate climates [14,20]. The fungus survives as microsclerotia and can persist in soils for over ten years [21]. Microsclerotia germinate to infect and colonize roots, subsequently invading the vascular system of susceptible hosts and causing disease symptoms. The pathogen is hemibiotrophic, and microsclerotia develop in diseased plants during and after host senescence. The disease cycle is completed when microsclerotia are released into soil as infested plant debris decays.

The host range of *V. dahliae* is particularly large and includes over 200 plant species from at least 14 different families [20,22]. Historically, monocots were not considered hosts of *V. dahliae,* but it has been demonstrated that many plants, including several economically important grasses, can be asymptomatic hosts of the pathogen [23]. Despite the wide host range of *V. dahliae*, isolates of the pathogen can differ dramatically in their ability to cause symptoms on different hosts [16,24,25]. Differential reactions among *V. dahliae* isolates have been documented in cotton, olive, artichoke, tomato, lettuce, and mint [16,24,26,27,28,29,30,31,32]. In general, isolates of *V. dahliae* from mint typically cause severe symptoms when re-inoculated onto susceptible *Mentha* species and cultivars, but the same isolates usually do not cause severe symptoms in other hosts [33]. Similarly, isolates from other host species usually do not cause Verticillium wilt symptoms when inoculated onto mint [16,31,32]. However, there are notable exceptions [16,17,18,34,35], and isolates from mint have been shown to cause Verticillium wilt symptoms in skullcap (*Scutellaria lateriflora* L.), tomato (*Solanum lycopersicum* L.), Canada thistle (*Cirsium arvense* L.), and lamb’s quarters (*Chenopodium album* L.) [18,36], while isolates from other hosts can occasionally cause mild or temporary symptoms on *Mentha* spp. [37]. Although the appearance of symptoms is considered the ultimate manifestation of infection, *V. dahliae* isolates from mint can asymptomatically infect and colonize the roots and stems of other species, including non-hosts and weeds, though the degree of colonization is often to a lesser extent in asymptomatic hosts [38,39]. Further complicating matters, it has been proposed that the aggressiveness of isolates may change over time [18,34,40,41], but more research is needed to understand the nature and extent of evolution in *V. dahliae* populations affecting agroecosystems. 

The general mechanisms of pathogenicity for *V. dahliae* have been reviewed previously [42]. Briefly, colonization of the xylem by the fungus results in vessel occlusion (either by the pathogen or as a host defense response), reduced transpiration, and eventually wilt [42,43,44], although cell-wall-degrading enzymes, manipulation of host defenses, and phytotoxins have been identified as additional potential pathogenicity factors in *V. dahliae* [42,45]. The specific mechanisms by which this occurs—and the relative importance of various factors—remains essentially uncharacterized in mint specifically.

## 4. Vegetative Compatibility and Genetic Diversity of *V. dahliae* Populations from Mint

Historically, isolates of *V. dahliae* have been classified according to vegetative compatibility group (VCG) based on their ability to undergo hyphal anastomosis with standardized nitrate (*Nit*) non-utilizing mutant tester strains [46,47,48]. VCGs provide a basis for self- and non-self-recognition that regulates heterokaryon formation, the parasexual cycle, and the transmission of virulence factors and mycoviruses [49]. There are at least four primary VCGs among *V. dahliae* isolates, with several being further divided into groups and subgroups based on the frequency and vigor of complementation [48,50,51,52,53,54,55,56]. To date, most *V. dahliae* isolates collected from symptomatic mint in the U.S. have been characterized as VCG2B [16,17,57]. VCG2B isolates obtained from other hosts are typically not as aggressive on mint, suggesting the presence of a mint-adapted lineage within VCG2B [16,31,34,37].

While VCGs have been tremendously useful in characterizing *V. dahliae* isolates and populations, the application of molecular tools has provided new insights into the genetic diversity of the pathogen and the relationships among VCGs [56]. This is especially true for VCG2B. Multiple studies using sequence analyses of the intergenic spacer region and housekeeping genes [55,56,58], amplified fragment length polymorphisms [55], microsatellite haplotyping [37], single nucleotide polymorphisms ([56], and genotyping-by-sequencing [59] indicate VCG2B is polyphyletic in origin. The relatively few molecular phylogenies that focused on or included mint isolates indicate that mint-adapted isolates are genetically distinct from other *V. dahliae* isolates [37,57,58,60,61].

Despite the pathogenic, VCG, and genetic diversity that has been documented among *V. dahliae* isolates, most studies report relatively low genetic diversity among *V. dahliae* populations. Anastomosis, hybridization, and recombination events have been documented or inferred in populations of *Verticillium*, including *V. dahliae*, but the structure of contemporary *V. dahliae* populations is highly clonal in nature [59,62,63,64,65]. Several studies using various molecular markers have reported low genetic diversity in *V. dahliae* isolates from mint [37,57,58,66], also indicative of a clonal reproductive model. Dung et al. [37] reported that a single microsatellite haplotype was predominant (92 out of 104 isolates) among a historical collection of mint isolates collected from mint in Washington, Oregon, Montana, Indiana, and Michigan. A follow-up study on contemporary populations in Oregon using genotyping-by-sequencing identified the presence of a predominant genetic group, which was also detected among isolates collected in Washington State, California, Montana, and Indiana [57]. Collectively, these results support the hypothesis that a mint-adapted, VCG2B lineage of *V. dahliae* is associated with U.S. mint production, and that it was probably distributed throughout the U.S. as the industry moved to new areas in an effort to escape the pathogen [16].

## 5. Interactions between *V. dahliae* and Root Lesion Nematodes in Mint

A variety of plant pathogenic nematodes can be recovered from soils associated with mint production, but most do not cause significant damage to mint or occur in large enough populations to cause concern. Of those that do, Pratylenchus, Longidorus, and Meloidogyne have been documented to cause damage or yield reductions to mint, and Pratylenchus, specifically *P*. *penetrans* and *P. neglectus*, are probably the most common nematode species associated with stand declines [67].

Both *P*. *penetrans* and *P. neglectus* have large host ranges and are widely distributed in the United States [68]. As endomigratory parasites, these nematodes penetrate roots resulting in water-soaked lesions. Brown lesions associated with feeding and migration cause root necrosis, girdling, and structural and physiological damage. Consequently, root function is compromised, resulting in stunted plants and thin stands. Damaged roots can leave infected plants prone to diseases caused by other soilborne pathogens.

Relationships and interactions between *V. dahliae* and Pratylenchus species have been documented in several crops, including peppermint and Scotch spearmint. For example, Wheeler et al. [69] used a machine-learning approach to conclude that the presence of Pratylenchus spp. was a significant predictor of Verticillium wilt foci in commercial mint stands. Co-infection of peppermint by *V. dahliae* and *P*. *penetrans* resulted in Verticillium wilt symptoms appearing two weeks earlier compared to infection by *V. dahliae* alone [70], and increased Verticillium wilt severity due to co-inoculations has been documented in both peppermint [31] and Scotch spearmint [71]. Negative additive and synergistic effects on foliar and root growth have also been observed [70,71], and *V. dahliae* colonization can increase in mint stems when plants are infected by *P. penetrans*. [31]. These additive or synergistic effects are often more pronounced when levels of *V. dahliae* inoculum are low. Interactions between *V. dahliae* and *P. penetrans* have not been observed in native spearmint [31].

Interactions between *V. dahliae* and *P. penetrans* on mint appear to be limited to the VCG2B mint pathotype of *V. dahliae*. Johnson and Santo observed increased disease severity and *V. dahliae* recovery when peppermint and Scotch spearmint were co-infected with *P. penetrans* and the VCG2B mint pathotype of *V. dahliae*, but not when they co-infected mint with the VCG4A potato pathotype of the fungus [31]. Similar, results were observed on potato, where the VCG4A potato pathotype was shown to interact synergistically with *P. penetrans* but VCG4B isolates did not [72]. The nature and mechanism of these markedly different reactions between *V. dahliae* isolates and *P. penetrans* on these hosts is not entirely clear.

While the most important interaction between nematodes and *V. dahliae* on mint involves *P. penetrans*, another root lesion nematode (*P. neglectus*) has been reported to interact with *V. dahliae* on peppermint [73,74,75]. As in the case of *P. penetrans*, co-inoculation with *V. dahliae* and *P. neglectus* decreased the incubation period required to detect Verticillium wilt symptoms and increased both the incidence and severity of wilt in peppermint plants compared to *V*. *dahliae* alone. The negative effects on root and foliage growth were also more severe with the combined presence of both pathogens. The impacts on incubation period, disease incidence and severity, and plant growth were proportional to the number of nematodes present, suggesting a significant role of nematode density on the magnitude of the interaction.

The mechanisms underlying the observed interactions between *V. dahliae* and root lesion nematode are not entirely understood. It has been hypothesized that wounds caused by root lesion nematode feeding can serve as infection courts for *V. dahliae*. However, several studies suggest that other mechanisms are involved. Bergeson [70] found that significant interactions between *V. dahliae* and *P. penetrans* could only be induced when the root-lesion nematode was introduced prior to *V. dahliae* infection; when the two pathogens were co-inoculated at the same time the same effect could not be reproduced. In another study, a double root technique was used to inoculate spatially separated roots of the same peppermint plant with *V. dahliae* and *P. neglectus*, resulting in a reduced incubation period and increased Verticillium wilt incidence and severity [75]. Finally, Bowers et al. [76] observed that initial infection by *V. dahliae* typically occurred at the root tip and in the zone of elongation, and the location of *V. dahliae* infection was not influenced by the presence of *P. penetrans*. On the other hand, they did observe increased infection at the root tip by *V. dahliae* in the presence of nematodes, but fungal infection courts were not associated with nematode feeding sites. Together, these results suggest that infection by *P. penetrans* changes the physiology of the host to confer increased susceptibility to *V. dahliae*, as opposed to simply providing wounds for infection courts. It is also possible that reproduction of *P. penetrans* is enhanced on hosts infected by *V. dahliae* [77]

## 6. Epidemiology of Verticillium Wilt

Verticillium wilt of mint is a monocyclic disease, in that the initial inoculum (in the case of *V. dahliae*, microsclerotia) that is present when the crop is planted is the primary source of inoculum for infections in the current season, and only one infection cycle per growing season is recognized. Microsclerotia of *V. dahliae* can be found in soil, infested plant debris, reused or contaminated irrigation water, and infected rhizomes used as planting stock [78,79]. Microsclerotia are stimulated to germinate by the presence of plant root exudates or other stimuli, including contact with plant debris [80,81]. Mint plants may show initial symptoms of Verticillium wilt throughout the growing season because infections can occur at various times during the growing season as roots grow and contact microsclerotia in the soil profile. The disease threshold can be as low as 10 microsclerotia/g of soil for peppermint [82] but more research is needed to identify the inoculum levels and thresholds that cause wilt in other *Mentha* species and cultivars.

In addition to microsclerotia, *V. dahliae* can produce asexual spores called conidia. Conidia of *V. dahliae* are hyaline, cylindrical to oval, and terminally produced in gleoid masses on short, verticillate conidiophores. Isolates from mint readily produce conidia on artificial media, and sporulation has been observed on infected and infested mint debris and on the debris of other plants [34]. Although *V. dahliae* has been called a “feeble saprophyte” [80], the pathogen can grow saprophytically and sporulate in uncropped soil under favorable conditions [80]. Such saprophytic growth and sporulation could potentially increase the production of microsclerotia and subsequent inoculum levels, but mycelia and conidia of *V. dahliae* do not survive for more than a few weeks in fallow soil [80,83,84]. Consequently, neither conidia nor mycelia are considered to serve as primary or secondary inoculum, nor survival structures, of the pathogen in mint production systems [80,85].

Many Ascomycetes undergo a sexual reproduction cycle to produce ascospores. Ascospores of plant pathogens can serve as inoculum or aid in their survival. *Verticillium dahliae* is considered to be heterothallic [86] and would require isolates of two different mating types (*MAT1-1* and *MAT1-2*) for sexual reproduction to occur [87]. Although both mating types have been documented in populations of *V. dahliae* [86,88], the fungus is only known to produce asexually and has no known teleomorph despite its broad host range and distribution [20]. The *MAT1-2-1* idiomorph is most often observed in populations of the pathogen [59,89], including isolates from mint [37,57]. The seemingly rare nature of *MAT1-1* isolates limits the likelihood for sexual reproduction in populations of *V. dahliae* from mint.

Verticillium wilt is also considered a polyetic disease, since inoculum can increase in field soils from one season to the next, and a progressive increase in Verticillium wilt incidence and severity is often observed in infested fields over succeeding years [69,85]. The buildup of inoculum from season to season is likely from the formation of microsclerotia in infected mint stems and rhizomes, coupled with their long survival in the soil [21]. Fields that are heavily contaminated with the wilt fungus are usually not suitable for production of peppermint and Scotch spearmint without soil fumigation or other management practices.

Both soilborne inoculum of *V. dahliae* and the resulting Verticillium wilt foci are usually aggregated and unevenly distributed within commercial production fields [90]. As time progresses, the number of disease foci often increase in number and size in perennial stands [85]. Disease clusters often occur along rows rather than between them, indicating the spread of inoculum through the distribution of soil during cultural practices. Soilborne inoculum can also be distributed throughout a field via irrigation, precipitation, and runoff [91]. As the stand ages, disease foci can coalesce, leading to reduced yields, large areas of mortality, weed problems, and early abandonment of the crop. The annual production of microsclerotia in stems of infected plants also contribute to the spread and increase in Verticillium wilt foci in older fields.

Long distance movement of *V. dahliae* can potentially occur through the transport of infected rhizomes used for establishing new fields [85]. It is also possible for the pathogen to be carried in any infested soil that might accompany field-produced rhizomes, as has been documented in potato seed tubers used for planting [92]. It was hypothesized that the original 1924 outbreak described in Mentha, Michigan was caused by infected propagative materials [6], and several instances of Verticillium wilt have been associated with infected rhizomes. For instance, a wilt outbreak was observed in a 1955 mint field in eastern Washington after planting rhizomes that were received from Indiana [4]. Another report, in 1961, cited the simultaneous appearance of the disease in eight fields in central Oregon, again likely due to the movement and planting of infected rhizomes [4]. Aggregated and clustered spatial patterns of Verticillium wilt in first-year mint fields with no prior history of mint production can be caused by infected planting material [11,85], and large wilt foci can often be found at field entrances or where rhizomes were piled prior to planting (Figure 2F).

## 7. Integrated Disease Management of Verticillium Wilt

The perennial aspect of U.S. mint production, coupled with the soilborne, polyetic nature of *V. dahliae*, makes controlling Verticillium wilt in mint a challenge. Existing methods of controlling Verticillium wilt in mint once the fungus has been introduced into a field are not satisfactory, practical, or economical, so exclusion, avoidance, and sanitation are of primary importance for long-term Verticillium wilt management. Once established, an integrated disease management approach that includes the management of root lesion nematodes, cultural practices that reduce the spread of the fungus and promote disease suppressive soils, and the use of less susceptible cultivars can reduce primary and secondary losses due to Verticillium wilt.

### 7.1. Exclusion, Avoidance, and Sanitation

The use of disease-free planting materials can prevent the introduction of *V. dahliae* into a new field and new mint-producing regions. Mint is vegetatively propagated and rhizomes used for commercial plantings in the U.S. start as tissue culture plantlets or as disease-free greenhouse grown stock [1]. Rhizomes are typically increased in the field prior to planting for commercial production, a necessary practice but one that can result in the introduction of *V. dahliae* into propagative materials. Rootstock should be propagated in clean soil in greenhouses, or in fields that have not been previously used to grow mint and/or have been treated with a soil fumigant. Reused irrigation water that may have been contaminated with *V. dahliae* should not be used to water the crop. Some states offer inspection and certification services for mint rhizomes intended for commercial plantings, a program aimed at reducing the spread of weeds, pests, and diseases, including *V. dahliae*. Although many mint rootstock certification programs often express a zero-tolerance for Verticillium wilt in planting stock, planting materials can be produced under less strict land requirements and carry an acknowledged risk of undetected *V. dahliae*.

Whenever possible, commercial plantings should be in fields that do not have a prior history of Verticillium wilt of mint or in fields that have not previously been cropped to mint [93]. If the field has a history of mint production and wilt, soil sampling prior to planting can be performed to determine the levels of both *V. dahliae* and *P. penetrans* in a field and decide if pre-plant management practices should be implemented to reduce the impact of Verticillium wilt in future years. However, soil sampling alone does not differentiate the mint-aggressive strains of *V. dahliae* from other strains, which may be present if the field has been previously planted to other hosts of *V. dahliae* such as potato or tomato.

If already present, reducing the amount of initial inoculum of *V. dahliae* in the soil before planting is essential for managing the monocyclic phase of the disease. The control of Verticillium wilt with the use of soil fumigation can be difficult due to the soilborne nature of the fungus and the perennial aspect of mint production in the U.S. There are fumigants that are effective against *V. dahliae* (metam sodium or 1,3-dichloropropene with or without chloropicrin), and many provide the additional benefit of nematode reduction, but these chemicals are expensive and need to be applied before planting mint [93,94]. Fumigation is expensive, subject to increasing environmental legislation, and may be either cost-prohibitive or impractical in the future, so other practices will likely be necessary for sustainable mint production moving forward.

Limiting the rate of disease spread after planting is essential in managing the polyetic phase of Verticillium wilt. Machinery can distribute the pathogen within rows during harvest and cultivation, leading to new infection foci, so limiting tillage and cultivation activities can reduce the spread of microsclerotia within a field [95]; however, this may not be possible in perennial stands of mint, which often require an annual cultivation to stimulate new growth prior to overwintering. Conversely, soil inversion and deep plowing of muck soils to a depth of 0.7 to 0.8 m can reduce Verticillium wilt through the burial and displacement of microsclerotia that were previously on or near the soil surface [96]; however, this practice is not possible in all mint producing regions. It cannot be emphasized enough that cleaning and sanitizing equipment and machinery used to cultivate, harvest, or transport mint can minimize spread within and among fields.

Under favorable conditions, *V. dahliae* can grow saprophytically on a variety of rotation crop and weed debris [34]. Debris of peppermint, weeds, and non-hosts has been demonstrated to be suitable substrates for the growth of *V. dahliae* isolates from mint, so effective crop debris management can help to limit the increase and persistence of the fungus over time [34]. Much of the aboveground growth is removed for distillation but cutting and harvest operations can leave infested mint stems in the field, where they eventually decompose and release microsclerotia.

Propane flaming can be performed after harvest to kill microsclerotia in aboveground stems and debris remaining in the field, thereby limiting the annual increase in microsclerotia levels and reducing the amount of primary inoculum available in the subsequent season [19,95,97]. In order to provide adequate control, internal stem temperatures must reach 60 °C or higher to kill the majority of *V. dahliae* propagules in infected stems. Tractor speeds of 3.2 to 4.3 kph were reported to kill over 99% of the fungus in infected stems and also incinerated infested debris, while speeds ≥ 4.8 kph were not as effective [95,98]. Soil temperatures are not significantly changed with this treatment, so the pathogen can survive propane flaming in belowground plant parts [95,99]. Site-specific flaming [99], coupled with aerial imaging and global positioning system (GPS) technology, could potentially be used for the targeted control of *V. dahliae* and prevent the increase and spread of wilt in young fields or those with low amounts of wilt overall.

### 7.2. Crop Rotation

Crop rotations generally do not result in satisfactory reductions in *V. dahliae* levels in soil and, in general, crop rotation by itself is not considered an effective control tactic due to the long survival and wide host range of *V. dahliae*. [100]. *V. dahliae* has been observed to colonize, infect, and reproduce on a wide range of crops and weeds, sometimes asymptomatically [18,38,81,101]. Specifically, isolates from mint have been shown to infect the roots of alfalfa (*Medicago sativa* L.), dry bean (*Phaseolus vulgaris* L.), red clover (*Trifolium pratense* L.), sudangrass (*Sorghum drummondii* Nees ex Steud.) and sweet corn (*Zea mays* L.) and the roots and stems of Austrian winter pea (*Pisum sativum* L.), barley (*Hordeum vulgare* L.), mustards (*Brassica juncea* L. and *Sinapis alba* L.), eggplant (*Solanum melongena* L.), pepper (*Capsicum annuum* L.), potato (*Solanum tuberosum* L.), and wheat (*Triticum aestivum* L.) to some degree [34,38,102,103].

The long survival of microsclerotia in soil requires the development of crop rotations that serve to reduce inoculum levels. Certain green manure crops have been shown to reduce the impact of Verticillium wilt in other crops [104]. Sudangrass, corn, and several Brassica species, particularly broccoli, have been shown to suppress Verticillium wilt symptoms or reduce inoculum levels [105,106,107]. The efficacy of green manures may be due to the production and release of glucosinolates, increased organic matter, changes in physical or chemical soil properties, and/or shifts in microbial diversity, composition, or activity in the soil during their growth and decomposition [108,109,110,111]. Although rotation and incorporation of certain green manures have been shown to be effective at reducing *V. dahliae* inoculum, more long-term studies are needed, especially considering the potential for the pathogen to infect and multiply on commonly grown green manure crops [38,101]. Unfortunately, rotating out of a profitable crop for a green manure may not always be a viable option for economic, logistical, or other reasons.

### 7.3. Host Resistance

Wilt-resistant cultivars, coupled with disease management practices that reduce the rate of disease development and prevent the accumulation of inoculum, offer potential for managing the polyetic stage of the disease. In some cases, such as in tomato and lettuce, host resistance can be attributed to cultivar and race-specific interactions [26,112,113,114]. Race 1 isolates of *V. dahliae* possess a gene (*Ave1*) that encodes a virulence factor, which is recognized by race 1-resistant cultivars of tomato bred to possess *Ve1*, an immune receptor-encoding gene [113,114]. It has also been found that some lettuce cultivars possess homologs of *Ve1* that confer resistance to race 1 isolates of *V. dahliae* [26,112]. However, race 2 isolates of *V. dahliae* lack *Ave1* and can cause Verticillium wilt symptoms on race 1-resistant cultivars [113]. Race 2 appears to be the most common *V. dahliae* race globally [89]. So far, all of the *V. dahliae* isolates from Mentha spp. in the United States have been characterized as race 2 [57], which suggests that other sources of resistance are required to manage Verticillium wilt in U.S. mint production systems.

Wilt resistance in mint does not completely prevent the fungus from colonizing the plant, but it does appear to limit fungal colonization or reproduction in the xylem and aboveground stems [17,23,115]. Lacy and Horner [23] observed extensive colonization and infection of roots in native spearmint and *M. suaveolens* by *V. dahliae* despite their resistance to the disease. However, overall pathogen recovery and fungal CFUs are usually reported to be much lower in resistant rather than susceptible mints [17,31,35,115]. Resistance in perennial mints can also be expressed as asymptomatic regrowth and recovery following initial infection, which may be an important resistance trait in mint [17,116,117,118]. Although symptoms are generally not as severe in resistant cultivars, yield reductions can occur even when symptoms are apparently mild [35,82].

Resistance to *V. dahliae* in other cultivated mints or mint accessions varies depending on cultivar or genetic background, *Verticillium* isolate, and inoculum level [17,119,120]. Plants can be effectively inoculated using root dips or soil drenches of conidial solutions or planting into naturally or artificially infested soil, and screening for resistance can be performed successfully in growth chamber (Dung, unpublished), greenhouse [17,121], and field settings [122]. Extending the duration of resistance screening through multiple cuttings or field seasons can provide valuable information about cultivar recovery and yield performance over time and under different environmental conditions [17,121,122], which can be particularly important for a perennial crop such as mint. Field trials to evaluate resistance require more time, effort, and resources, but their larger scale can allow for more effective evaluations of hay and oil yields over the course of several seasons and under more realistic production conditions [122].

Unfortunately, both peppermint and Scotch spearmint are sterile hybrids with a complex polyploid genome and cannot be bred for increased resistance to Verticillium wilt [123]. Native spearmint (*M. spicata* L.) is relatively resistant to the disease and can be planted in fields where peppermint or Scotch spearmint would not be productive. However, native spearmint oil has specific end uses and is not a replacement or substitute for peppermint or Scotch spearmint oil.

Since peppermint and Scotch spearmint are infertile hybrids, past development of Verticillium wilt-resistant cultivars relied upon non-conventional breeding methods, including cobalt 60 gamma irradiation-induced mutations, hybridization of fertile seed, polyploid clones, and transgenic approaches [117,121,124]. Several resulting cultivars of peppermint, including ‘Todd’s Mitcham’ and ‘Murray Mitcham’, exhibit varying levels of resistance to Verticillium wilt and are commercially grown [125,126]. However, these peppermint cultivars are not completely resistant and should not be planted in highly infested soils. Verticillium wilt-resistance exists in other *Mentha* mutants, hybrids, and fertile clones, but resistant cultivars must also possess the desired yield potential and oil characteristics that are required for economic production and marketing to end-users [121]. Currently, the number of commercially acceptable cultivars with appreciable resistance to Verticillium wilt is limited.

Genomic resources for Mentha are being developed to better understand the genetic basis of Verticillium wilt and oil biosynthesis, with the ultimate goal of producing Verticillium-resistant cultivars that yield commercially acceptable oils [2]. Research efforts in the U.S. have focused on the proposed progenitors of ‘Black Mitcham’ peppermint, *M. suaveolens* Ehrh., *M. aquatica* L., and *M. longifolia* (L.) Huds., some accessions of which have shown resistance to Verticillium wilt [127,128]. A study by Vining et al. [128] found that most accessions of *M*. *suaveloens* from the USDA National Clonal Germplasm Repository (Corvallis, OR, USA) were triploid or tetraploid and exhibited a high degree of resistance to Verticillium wilt, while accessions of *M. aquatica* and *M. longifolia*, which were octoploid or nonaploid and diploid, respectively, exhibited a range of resistance.

The self-fertile, diploid nature of *M. longifolia* makes it an amenable species for genetic research in Mentha [127]. Verticillium wilt-resistant accessions of *M. longifolia* have been described [17,127], and putative orthologs of Ve1 have been identified in the *M. longifolia* genome [119,120]. The mVe1 ortholog identified in *M. longifolia* codes for a leucine-rich repeat domain, a motif frequently observed in plant resistance proteins. The mVe1 gene product showed 51% amino acid identities with predicted proteins produced by tomato Ve1 and Ve2 genes and putative orthologs were also identified in *M. spicata* and ‘Black Mitcham’ peppermint. Unfortunately, transformation of 67 *M. x piperita* plants to express mVe1 or mVe2 did not result in the identification of any resistant phenotypes when evaluated in the greenhouse [124]; in hindsight, these results may have been due to a failure of the plants to recognize the race 2 *V. dahliae* isolates from mint that were used in the study. Additionally, the segregation of progeny from crosses of resistant and susceptible *M. longifolia* accessions indicated that the variation for resistance was polygenic. Together, these results suggest that quantitative traits will be required for effective and durable host resistance in mint cultivars.

## 8. Conclusions and Future Research

The first report of Verticillium wilt in mint was nearly a century ago [6], yet Verticillium wilt continues to be the most economically important disease affecting mint production in the United States. The perennial aspect of mint production, the vegetative propagation of the crop, and the soilborne nature of *V. dahliae* present difficult challenges towards Verticillium wilt management. Consequently, management efforts should employ a multifaceted approach, which begins with the use of certified, pathogen-free planting materials and sanitation to prevent the introduction and spread of the fungus. Green manures and specific crop rotations, when possible, can provide long-term benefits in addition to Verticillium wilt suppression. Aerial imaging, coupled with GPS-enabled devices and machinery, offer the potential for early detection and site-specific control of Verticillium wilt. The detection and management of root lesion and other mint pathogenic nematodes is also important for mint and other crops in U.S. mint production systems. While not covered in this review, the use of biological control agents and practices that promote disease suppressive soils have the potential to manage *V. dahliae* in ways that are just beginning to be understood [109,129,130,131,132,133]. 

The availability of molecular resources, including genomic sequences of *V. dahliae* [134,135] and *M*. *longifolia* [123], will be invaluable for developing novel tools and strategies for Verticillium wilt control in mint moving forward, but many challenges remain. DNA-based methods have been developed to detect and quantify *V. dahliae* in soil samples with improved sensitivity and selectivity, but these methods require additional context in order to be meaningful for growers (i.e., how do the results correspond to traditional plating and how do they translate to wilt risk?). Despite the clonal, asexual nature of the pathogen, identifying molecular markers that can be used for the rapid and reliable detection of the mint-adapted VCG2B strains has been challenging (Dung, unpublished). Long-term, there is a need for the development of genomic resources towards the goal of breeding high-yielding, wilt-resistant cultivars with desirable oil qualities. Hopefully, continued research on Verticillium wilt in mint and other crops will produce effective, durable, and economical approaches to manage this important disease.
